# Drivers of Palliative Care and Hospice Use Among Patients With Advanced Lung Cancer

**DOI:** 10.1002/cam4.70518

**Published:** 2025-01-17

**Authors:** Megan C. Edmonds, Melissa Mazor, Mayuri Jain, Lihua Li, Marsha Augustin, José Morillo, Olivia S. Allen, Amina Avril, Juan P. Wisnivesky, Cardinale B. Smith

**Affiliations:** ^1^ School of Public Health Virginia Commonwealth University Richmond Virginia USA; ^2^ Division of General Internal Medicine Icahn School of Medicine at Mount Sinai New York New York USA; ^3^ Department of Population Health Science and Policy Icahn School of Medicine at Mount Sinai New York New York USA; ^4^ Albert Einstein College of Medicine Bronx New York USA; ^5^ Population Health Science & Policy Icahn School of Medicine at Mount Sinai New York New York USA; ^6^ Divisions of Hematology and Medical Oncology and the Tisch Cancer Institute Icahn School of Medicine at Mount Sinai New York New York USA

**Keywords:** ethnic and racial minorities, hospice care, lung cancer disparities, palliative care

## Abstract

**Purpose:**

Despite rigorous evidence of improved quality of life and longer survival, disparities in the utilization of palliative and hospice care persist for racial and ethnic minority patients with cancer. This study evaluated the impact of psychosocial factors on utilization of these services.

**Methods:**

Patients with advanced lung cancer were recruited at a large academic urban hospital. Patients were surveyed about their knowledge of palliative care and hospice and their beliefs regarding medical mistrust, lung cancer care, palliative care and hospice. We used univariate and multivariable logistic regression analyses to examine the association between mistrust, knowledge and beliefs among the entire cohort and minority (Black and Hispanic) and non‐minority patients on utilization of palliative care consultation and hospice care use.

**Results:**

Ninety‐nine of the enrolled participants had a mean age of 64 years. Minority patients were more likely to receive a palliative care referral (*p* < 0.001) and attend a consult (*p* = 0.003). Similarly, they were more likely to receive a hospice referral (*p* = 0.04), however there was no difference in hospice care use based on minority status (*p* = 0.102). In our adjusted model, older patients and those reporting negative lung cancer beliefs were more likely to receive hospice care (OR: 1.06, 95% CI: 1.004–1.138; OR: 1.04, 95% CI: 1.002–1.093, respectively).

**Conclusion:**

Minority patients with advanced lung cancer were more likely to receive a palliative care referral and specialty level consultation when compared to non‐minority patients. Our work highlights the importance of proactive referral processes in facilitating access to palliative and hospice services, particularly among younger patients.

## 1 Introduction

In 2020, there were approximately 602,400 lung cancer deaths [1, 2, 3]. Despite treatment advancements, lung cancer remains the leading cause of cancer mortality in the United States and is commonly diagnosed at more advanced stages [1, 2]. Unfortunately, the burden of lung cancer disproportionately impacts racial and ethnic minorities [1, 2, 4]. The incidence of cancer is not only higher in Blacks than Whites, but is increasing at a faster rate; 1.2% versus 0.8% per year [5]. Minority patients with advanced lung cancer more frequently have higher baseline and persistent supportive care needs relative to non‐minority patients throughout the trajectory of illness [6]. Moreover, minority patients are less likely to receive high quality cancer care, are less likely to receive care aligned with stated preferences, and have higher utilization of acute care services at the end of life [6, 7, 8].

Palliative care, in conjunction with standard oncologic treatment, is advocated for patients diagnosed with advanced cancer, aiming to enhance quality of life, disease management, and address the multifaceted supportive care needs of both patients and their families [9]. Evidence supports the integration of specialty palliative care in routine oncologic care among patients with advanced lung cancer with a demonstrated benefit of two‐month overall survival and improved quality of life compared to standard oncologic care alone [10, 11]. Despite recommendations, racial and ethnic disparity in accessing these services persist [7, 12]. Similarly, hospice care, recognized for its role in end‐of‐life care and associated quality‐of‐life improvements, remains underutilized among racial and ethnic minority populations [13]. The decision‐making process of cancer patients regarding the utilization of palliative and hospice care is significantly influenced by their psychosocial factors such as their beliefs, culture and understanding of their illness and treatment options [14, 15, 16]. Prior works supports that However, there is a notable dearth of research exploring the specific beliefs guiding the utilization of these services among patients with advanced cancer. Previous studies have indicated negative beliefs and misconceptions regarding palliative and hospice care, particularly among patients with lung cancer [17, 18, 19].

To better understand drivers of disparities of palliative and hospice care use, we used data from a prospective cohort of newly diagnosed patients with advanced lung cancer. The study aims to compare the impact of knowledge, sociocultural, and illness beliefs on receipt of palliative and hospice care among minority and non‐minority patients with advanced lung cancer. We hypothesized that relative to non‐minority patients, minority patients will hold more negative beliefs toward palliative and hospice care which contributes to underutilization of these services.

## 2 Methods

### 2.1 Study Design and Data Collection

**FIGURE 1:**
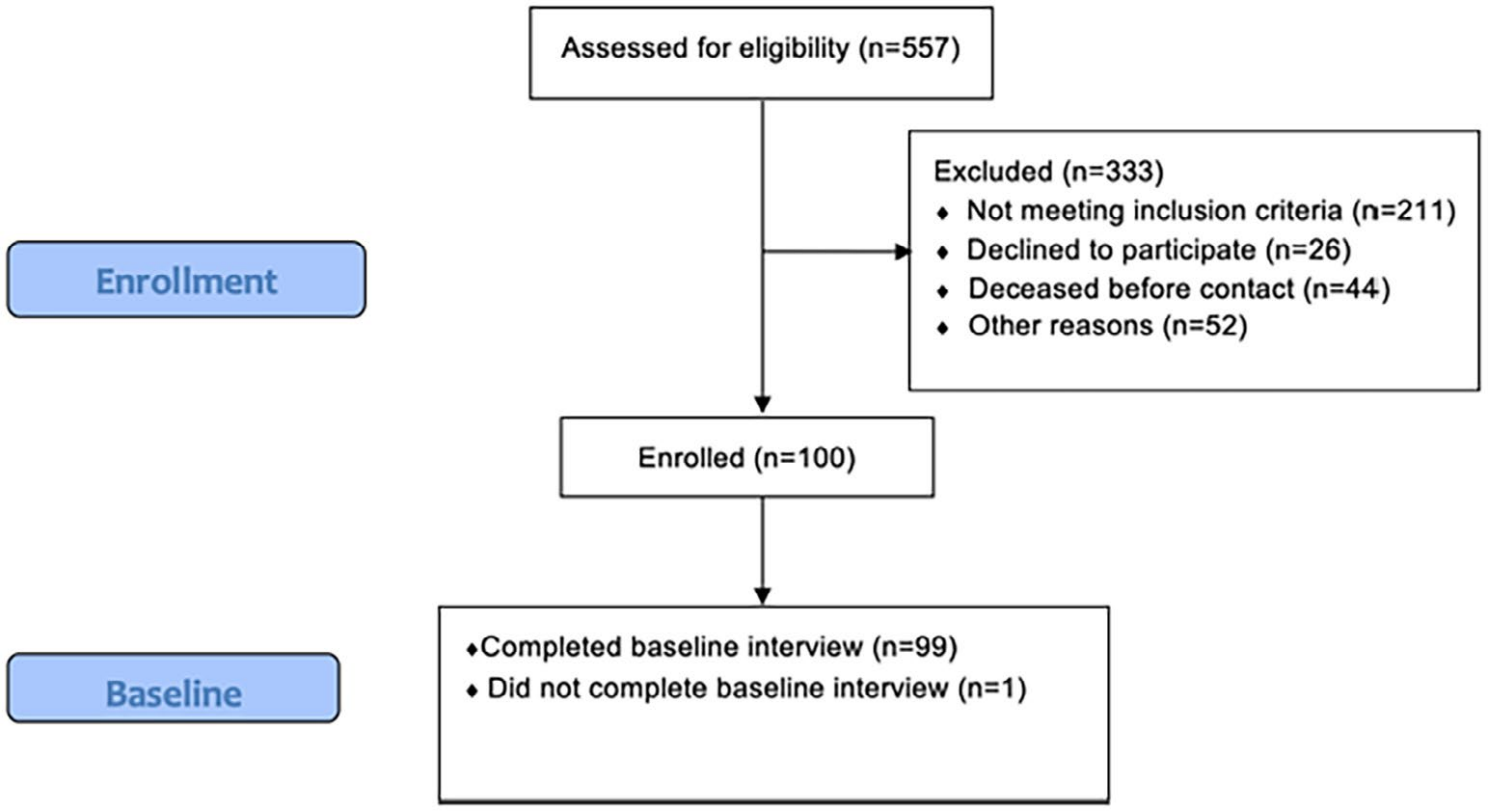
Study flow diagram.

We enrolled patients with newly diagnosed advanced (stage III and IV) lung cancer diagnosed at our institution between March 2014 and June 2018. Patients were enrolled in the study if they met the following criteria: (i) diagnosis ≤ 3 months with stage III or IV, non‐small cell lung cancer (NSCLC) or extensive stage small cell lung cancer (SCLC); (ii) ≥ 18 years of age; (iii) English or Spanish speaking; and (iv) had an available telephone contact number. Patients were excluded if they were either diagnosed or treated for another cancer within 5 years, or if they did not complete the baseline interview (Figure 1).

All eligible patients were enrolled from a single urban hospital in New York City, NY via trained clinical research assistants. Eligible patients provided informed consent and completed validated surveys at baseline and at, 4‐, 8‐ and 12‐months from enrollment. Surveys were conducted in person or telephonically by a research coordinator bilingual in English and Spanish. The full details of our recruitment procedures can be found in our prior publication [6]. The Icahn School of Medicine's institutional review board reviewed and approved this study (IRB #13‐00368).

### 2.2 Measures

#### 2.2.1 Outcome

The primary outcome was receipt of specialty palliative care consult and/or hospice care. Receipt of specialty palliative care consult was defined as being seen in a supportive oncology clinic as determined by documentation in the electronic health record (EHR) of a visit. Hospice care use (inpatient or outpatient) was abstracted from the EHR. Assessment of both outcomes were censored at the end of study follow‐up (i.e., 12 months from enrollment).

#### 2.2.2 Demographic Variables

Demographic variables included: age, sex, marital status, primary language (English and/or Spanish), education attainment (< high school, high school graduate or GED, college degree), and household income (≤ $30,000, $30,000–$50,000, > $50,000). Participants self‐reported racial and ethnic status which was categorized as minority (Black and/or Hispanic) and non‐minority (non‐Hispanic White). Clinical variables collected from the EHR included: Charlson comorbidity index [20, 21], Eastern Cooperative Group (ECOG) performance status [22], cancer type (non‐small cell lung cancer [NSCLC] versus small‐cell lung cancer [SCLC]), cancer stage, and treatment received (radiation and/or chemotherapy) [23].

#### 2.2.3 Patient Beliefs/Knowledge

Lung cancer beliefs were measured using the eight item Revised Illness Perception Questionnaire (IPQ), which assesses five domains: illness coherence, timeline, control, controllability or cure of the disease, perceived consequences of lung cancer and emotional representations of lung cancer such as anxiety, fear and worry about the disease (Cronbach's alpha 0.79–0.89) [24]. All items were measured on a 10‐point Likert scale. Scores across the 8‐item scale were summed with a range of (0–63) with higher scores indicating more negative illness perception toward lung cancer [e.g., “How long do you think your lung cancer will continue? (0=a very short time, 10=forever)”].

Palliative care beliefs were assessed using items adapted from the IPQ, newly created items, and selected questions from the Perceptions of Palliative Care Instrument (PPCI) [25]. We used 22‐items from this instrument that pertains only to the emotional and cognitive reactions to palliative care use on a 5‐point Likert scale, Cronbach's alpha = 0.67–0.93). Items were summed and reverse coded when needed with a range of (6–86). Higher scores across the 22 items indicate less negative palliative care beliefs (e.g., “Palliative care helps manage pain and other symptoms (e.g., shortness of breath, nausea).”

Hospice Beliefs were measured using 12 items from the Hospice Beliefs and Attitudes Scale (HBAS) (Cronbach's alpha = 0.74) [26]. Responses were on a 5‐point Likert scale, with a total score ranging from 9 to 48. Items were reverse coded when needed. Higher scores are indicative of less favorable hospice beliefs (e.g., “If I were dying, I would want hospice care”).

Medical mistrust was assessed using 10‐items from the 12‐item Group‐Based Medical Mistrust Scale (Cronbach's alpha = 0.83) [27]. The scale consists of three subscales: perceived discrimination, suspicion and disparities in healthcare settings and lack of support within a healthcare setting. Responses were on 5‐point Likert scale (strongly agree to strongly disagree), with scores ranging from 3 to 45. Items were reverse coded as needed when needed and summed. Higher scores reflect greater medical mistrust [27].

### 2.3 Processes of Care

We captured processes of care for our study outcomes using patients' EHR to determine if patients received a referral for hospice and/or palliative care, and to identify if a palliative care consult was scheduled.

#### 2.3.1 Palliative/Hospice Care Knowledge and Preferences

We assessed patients' general knowledge of palliative and hospice care with the question “How much would you say you know or have heard about palliative care?” Responses were on a 5‐point Likert scale. Additionally, we asked, “If there was a choice between hospice and palliative care today, which would you prefer?” Responses were palliative care, hospice, or neither.

### 2.4 Data Analysis

Characteristics of the sample were compared by minority status with respect to baseline sociodemographic factors, knowledge of palliative and hospice care and perception (palliative care, hospice care, lung cancer, and medical mistrust) constructs. Continuous variables were summarized by the mean and standard deviation or the median and interquartile range as appropriate, while categorical variables were summarized by frequency (*N*) and percentage (%). Distributions of continuous variables were compared by minority status using *t*‐test or Wilcoxon test, and categorical variables were compared using chi‐squared test or Fisher test. We performed a multivariable logistic regression to test the association between sociodemographic characteristics, beliefs and knowledge about lung cancer, and palliative care on palliative care and hospice care utilization. We adjusted for sex, age, marital status, income, Charlson comorbidity, medical mistrust, lung cancer belief scores, palliative/hospice care belief scores and palliative/hospice care knowledge. A power calculation for palliative care use outcome determined 83% power of an expected study sample of 100. A power calculation for hospice use outcome determined 80% power of an expected study sample of 100. All statistical analyses were conducted using SAS (Version 9.4. SAS Institute Inc., Cary). All the tests were two sided with *p* value < 0.05 considered as statistically significant.

## 3 Results

A total of 99 participants with advanced lung cancer were included in this study. The baseline characteristics, knowledge, beliefs and preferences are presented in Table 1. Overall, 55% (*n* = 55) of patients identified as minorities. Compared to non‐minorities, minority patients were less likely to be married (*p* < 0.001), had lower educational attainment (*p* < 0.001), were more likely to have Medicaid insurance (*p* = 0.003), had a lower household income (*p* < 0.001) and had a poorer performance status at baseline (*p* = 0.02). There was no difference in age, gender, religiosity or comorbidity index among the groups (*p* > 0.005 for all comparisons).

**TABLE 1:** Baseline characteristics, knowledge, beliefs and preferences of minority and non‐minority patients with advanced lung cancer.

Variable	Non‐minority *N* = 44, (%)	Minority *N* = 55, (%)	*p*
Age in years			
Mean ± SD	65.9 ± 9.7	63.2 ± 10.6	0.190
Self‐identified gender, female	30 (68.2%)	32 (58.2%)	0.310
Marital status			
Married	28 (63.6%)	15 (27.3%)	< 0.001
Language			
English	42 (95.4%)	45 (81.8%)	0.040
Spanish	2 (4.5%)	27 (49.1%)	< 0.001
Education			
< High school	0 (0%)	16 (29.1%)	< 0.001
High school graduate or GED	10 (22.7%)	15 (27.3%)	
College degree/professional training	32 (72.7%)	23 (41.8%)	
Unknown	2 (4.6%)	1 (1.8%)	
Religiosity/spirituality			
Very or somewhat religious/spiritual	28 (63.6%)	45 (81.8%)	0.070
Not very or not at all religious/spiritual	14 (31.8%)	10 (18.2%)	
Missing	2 (4.6%)	0 (0%)	
Primary insurance			
Medicare	23 (52.3%)	31 (56.4%)	0.003
Commercial	19 (43.2%)	10 (18.2%)	
Medicaid	2 (4.5%)	14 (25.4%)	
Household income			
≤ $30,000	4 (9.1%)	37 (67.3%)	< 0.001
$30,001–$50,000	5 (11.4%)	8 (14.5%)	
Over $50,000	31 (70.4%)	5 (9.1%)	
Unknown	4 (9.1%)	5 (9.1%)	
Charlson comorbidity index			
Mean ± SD	6.27 ± 2.45	6.65 ± 2.45	0.440
2 to 4	15 (34.1%)	11 (20%)	0.11
5 and above	29 (65.9%)	44 (80%)	
ECOG performance status			
0–1	39 (88.6%)	38 (69.1%)	0.020
2–4	5 (11.4%)	17 (30.9%)	
Patient beliefs			
Palliative care beliefs			
Mean ± SD	52.68 ± 13.37	52.23 ± 15.39	0.897
Hospice care beliefs			
Mean ± SD	29.36 ± 4.51	33.04 ± 6.10	0.001
Lung cancer beliefs			
Mean ± SD	29.45 ± 11.34	35.4 ± 15.0	0.031
Medical mistrust			
Mean ± SD	23.84 ± 4.93	26.4 ± 6.24	0.028
Palliative care	22 (50.0)	30 (54.6)	0.069
Hospice	3 (6.8)	1 (1.8)	
Neither	18 (40.9)	15 (27.3)	
Unknown	1 (2.3)	9 (16.3)	
Palliative care			
Some knowledge	26 (59.1)	11 (20)	< 0.001
None	16 (36.3)	43 (78.2)	
Unknown	1 (2.3)	1 (1.8)	
Hospice care			
Some knowledge	39 (88.6)	35 (63.6)	0.019
None	5 (11.4)	14 (25.4)	
Unknown	0 (0.0)	1 (1.8)	

Compared to non‐minorities, minority patients were less likely to have knowledge about palliative (78.2% vs. 36.3%; *p* < 0.001) and hospice care (25.4% vs. 11.4%; *p* = 0.019), had less favorable hospice beliefs (33.04 ± 6.10 vs. 29.36 ± 4.51; *p* = 0.001), more negative lung cancer beliefs (35.4 ± 15.0 vs 29.45 ± 11.34; *p* = 0.031) and had greater levels of medical mistrust (26.4 ± 6.24 vs. 23.84 ± 4.93; *p* = 0.028). There were no differences between minority and non‐minority patients with respect to palliative care beliefs. Overall, patients stated a preference for palliative care referral with no differences among minorities and non‐minorities (54.6% vs. 50%; *p* = 0.069).

Outcomes for referrals to and receipt of palliative care and hospice are in Table 2. Compared to non‐minorities, minorities had higher rates for receiving palliative care referral (63.6% vs. 31.8%; *p* < 0.001) and attending a palliative care consult (56.4% vs. 25%; *p* < 0.003). Similarly, minority patients had higher rates of hospice referral (47.3% vs. 27.3%; *p* = 0.04). However, there were no significant differences in receipt of hospice care (32.7% vs. 18.2%; *p* = 0.102).

**TABLE 2:** Palliative and hospice care referral and utilization among minority and non‐minority patients with advanced lung cancer.

Outcome	Non‐minority *N* = 44, (%)	Minority *N* = 55, (%)	*p*
Received palliative care referral	14 (31.8)	35 (63.6)	< 0.001
Attended palliative care consult	11 (25)	31 (56.4)	0.003
Received hospice care referral	12 (27.3)	26 (47.3)	0.04
Received hospice care	8 (18.2)	18 (32.7)	0.102

The association between knowledge, beliefs and preferences by palliative care and hospice utilization are in Table 3. Patients utilizing palliative care had less favorable beliefs toward hospice (*p* = 0.01) and higher negative lung cancer beliefs (*p* = 0.01). Among those utilizing hospice, there were more negative lung cancer beliefs (*p* = 0.043). There were no significant differences in mistrust, patient preferences or knowledge among those utilizing palliative care or hospice.

**TABLE 3:** Knowledge, beliefs and preferences of patients with advanced lung cancer by palliative and hospice care utilization.

Variable	Palliative care utilization		Hospice care utilization	
	Yes *N* = 42, (42.4%)	*p*	Yes *N* = 26, (26.3%)	*p*
Patient beliefs				
Palliative care belief				
Mean ± SD	55.26 ± (14.38)	0.1	56.0 ± (14.09)	0.143
Hospice care belief				
Mean ± SD	33.26 ± (5.30)	0.01	32.88 ± (4.61)	0.125
Lung cancer beliefs				
Mean ± SD	36.78 (14.53)	0.01	37.42 (13.49)	0.043
Medical mistrust				
Mean ± SD	26.40 ± (5.86)	0.09	26.35 ± (5.30)	0.27
Patient preferences for palliative versus hospice				
Palliative care	22 (52.4)	0.3	17 (65.4)	0.069
Hospice	0 (0)		0 (0)	
Neither	14 (33.3)		8 (30.8)	
Unknown	5 (11.9)		0 (0)	
Refused	1 (2.4)		1 (3.8)	
Patient knowledge about palliative care and hospice				
Palliative care		0.6		0.825
Some knowledge	13 (30.9)		9 (34.5)	
None	28 (66.7)		17 (46.1)	
Unknown	1 (2.4)		2 (2.7)	
Hospice care				
Some knowledge	13 (31.0)		19 (53.8)	
None	13 (21.)	0.16	17 (20.6)	0.087
Don't know	4 (9.5)		2 (2.7)	
Unknown	1 (2.4)		1 (1.4)	

In our adjusted multivariable logistic regression analysis (Table 4), adjusting for socio‐demographic characteristics and beliefs, patients who were older (odds ratio [OR]: 1.07; 95% CI: 1.00–1.14) and reported more negative lung cancer beliefs (OR: 1.05; 95% CI: 1.00–1.09) had significantly higher odds of receiving hospice care. No significant differences were found for other demographics or beliefs on use of hospice. In the adjusted association between race and the use of palliative care there were no significant differences found for any of the evaluated variables (Table 5).

**TABLE 4:** Adjusted association between sociodemographic characteristics, beliefs and knowledge about lung cancer, and hospice care on hospice utilization.

Variables	Odds ratio	95% CI	*p*
Race/ethnicity			
Non‐minority	Ref	Ref	Ref
Minority	2.536	0.568–11.312	0.2227
Gender			
Male	Ref	Ref	Ref
Female	1.656	0.553–4.961	0.3678
Marital status			
Unmarried	Ref	Ref	Ref
Married	2.691	0.714–10.412	0.1437
Household income			
≤ $30,000	Ref	Ref	Ref
$30,001–$50,000	1.183	0.230–6.083	0.8402
Over $50,000	0.969	0.195–4.814	0.9693
Unknown	0.296	0.034–2.582	0.2707
CCI cat			
5 and above	Ref	Ref	Ref
2–4	1.214	0.291–5.071	0.7902
Hospice care belief score	1.011	0.916–1.115	0.8227
Medical mistrust belief score	1.032	0.943–1.130	0.4944
Lung cancer belief scores	1.047	1.002–1.093	0.0394
Age	1.069	1.004–1.138	0.0383
Hospice care knowledge			
None	Ref	Ref	Ref
Some	1.585	0.371–6.762	0.5341
Unknown	5.128	0.592–44.385	0.1377

*Note:* Model controlled for sex, age, marital status, income, CCI cat, medical mistrust belief scores, lung cancer belief scores, hospice care belief scores, hospice care knowledge.

**TABLE 5:** Adjusted association between sociodemographic characteristics, beliefs and knowledge about lung cancer, and palliative care on palliative care utilization.

Variables	Odds ratio	95% CI	*p*
Race/ethnicity			
Non‐minority	Ref	Ref	Ref
Minority	1.88	0.525–6.737	0.3323
Gender			
Male	Ref	Ref	Ref
Female	1.053	0.390–2.847	0.9184
Marital status			
Unmarried	Ref	Ref	Ref
Married	0.921	0.310–2.735	0.882
Household income			
≤ $30,000	Ref	Ref	Ref
$30,001–$50,000	0.762	0.186–3.115	0.7046
Over $50,000	0.441	0.106–1.833	0.26
Unknown	0.802	0.159–4.034	0.7887
CCI cat			
5 and above	Ref	Ref	Ref
2–4	0.74	0.222–2.465	0.6242
Palliative care belief score	1.022	0.988–1.057	0.2114
Medical mistrust belief score	1.008	0.927–1.096	0.852
Lung cancer belief scores	1.022	0.984–1.061	0.2678
Age	0.993	0.941–1.047	0.7852
Palliative care knowledge			
None	Ref	Ref	Ref
Some	1.605	0.426–6.044	0.4844
Unknown	1.302	0.412–4.119	0.6532

*Note:* Model controlled for sex, age, marital status, income, CCI cat, medical mistrust belief scores, lung cancer belief scores, palliative care belief scores, palliative care knowledge.

## 4 Discussion

Although palliative and hospice care use are associated with improved quality of life and prolonged survival for patients with advanced cancer, minoritized patients often underutilize these services [13]. This study examined factors associated with palliative and hospice care use in a sample of minority and non‐minority patients diagnosed with advanced lung cancer. Our study sheds light on the disparities in palliative and hospice care utilization among patients with lung cancer, particularly highlighting the impact of beliefs and perceptions on access to these vital services. A significant proportion of minority patients were referred to specialty palliative care and hospice. While nearly half received the specialty palliative care consultation, hospice utilization rates remain suboptimal. Our findings underscore the need for targeted interventions to address beliefs surrounding lung cancer and hospice care, as well as the importance of proactive referral processes in facilitating access to palliative and hospice services, particularly among older patients.

Disparities in the uptake of palliative and hospice care are complex with conflicting data in the literature specifically about the utilization of specialty palliative care among minoritized individuals [12, 28, 29]. Our data demonstrate that minority patients were more likely to receive referrals and attend outpatient palliative care consults compared to their non‐minority counterparts. Our findings significantly add to existing literature as previously higher rates of inpatient, but not outpatient palliative care, have been identified among minoritized individuals [30, 31, 32, 33]. One possible explanation of our unique findings could be related to physician perceptions of discord in discussions with minority patients and their families, as evidenced by previous studies documenting differential communication patterns based on patient race [34]. Physicians may also perceive greater discord and anticipate reluctance to have serious illness conversations among minority patients, leading them to seek assistance through palliative care consultations. Factors such as physician implicit bias may also contribute to an underlying barrier to patient‐centered communication, thus, increasing the chance that minorities receive care that is not standard or might be discordant with preferences and values [35, 36, 37]. Additional physician‐related factors include a lack of communication skills training and perceived ability among oncologists to have emotionally charged conversations contributing to higher likelihood for referral [36].

Additionally, minority patients were more likely to be referred to hospice. It's possible that physicians may view the additional services provided by hospice as especially beneficial for minority patients, whom they may perceive as having limited resources compared to their White counterparts. In fact, Black individuals with cancer have reported a greater perceived need for certain hospice services (home health aide, visiting nurse, etc.) than White individuals [38]. However, despite the higher rates of referral to hospice, our findings also highlight a concerning trend of underutilization of hospice among minority patients even when being referred, suggesting that barriers beyond referral persist in accessing end‐of‐life care services.

We observed significant disparities in knowledge levels and beliefs related to lung cancer, palliative care and hospice between minority and non‐minority patients. Studies have consistently shown that the proportion of adults who have knowledge of palliative and hospice care is low, with few focusing specifically on minoritized individuals with advanced cancer [39, 40, 41]. Our data expand this literature as individuals in our study demonstrated lower levels of knowledge about palliative and hospice care. These knowledge gaps can stem from a multitude of sources, including limited access to healthcare resources and systemic barriers within the healthcare system. Minority patients, in particular, may face unique challenges in accessing information about palliative and hospice care due to language barriers, mistrust of the healthcare system, and cultural taboos surrounding end‐of‐life discussions.

Among the entire cohort, patients with less favorable beliefs about hospice and lung cancer were more inclined to utilize palliative care. This association may be due to the fact that patients are feeling less hopeful about their cancer treatment and side effects and are wanting supportive care but not ready to consider hospice at that time [42, 43, 44]. Despite lack of statistical significance in the adjusted model potentially due to small sample size, this finding underscores the importance of specialty palliative care as a preferred modality to provide supportive care to those with advanced cancer. Moving forward, future research should prioritize understanding and addressing facilitators and barriers to palliative care referral among minoritized persons. While newer research exists evaluating models of care delivery that is tailored based on level of patient need, they often exclude or underreport minoritized populations [11, 45]. Focusing on research that includes a diverse range of participants and addresses the unique beliefs and perceptions of these populations is crucial for achieving more equitable outcomes in palliative care delivery. Furthermore, improving access to these essential services requires multifaceted interventions including policy initiatives that focus on value‐based care and educational initiatives that improve patient‐centered communication within the oncologic health setting [35, 46].

Patients with cancer underutilize hospice; when they do enroll, they enroll late, often less than 7 days before death, and have a shorter length of stay than non‐cancer patients. Collectively, these influencing factors diminish the positive impact of hospice [47]. We found that older patients and those who held more negative lung cancer beliefs were significantly more likely to utilize hospice. This supports other data that suggest uptake and adherence to recommended medical care and treatment modalities is a function of one's emotional and risk perception of their own illness [16, 48]. Therefore, future interventions aimed at improving attitudes toward illness, addressing misconceptions, and providing value‐based care tailored to individual emotional needs may play a crucial role in promoting timely and appropriate utilization of hospice care among patients with advanced lung cancer.

## 5 Limitations

This study used a cross‐sectional study design which limits our ability to determine causation. Thus, our sample size of 99 limits a more robust analysis of predictors of palliative and hospice care utilization. Additionally, hospice use was confirmed using the EHR, which may not capture all patients receiving hospice care at home. However, our rates of hospice utilization are higher than reported in the literature thus we believe this to be an accurate representation. Lastly, while we were able to examine the role of physician referrals for palliative/hospice care, we did not examine the role of patient‐provider communication which may influence receipt of hospice and/or palliative care. Despite these limitations our study presents strengths in a novel research question about the receipt of palliative and hospice care among patients with lung cancer; we have highlighted important aspects of cancer care delivery that help facilitate optimal uptake of these services among minoritized patients.

## 6 Conclusion

Overall, our study highlights the complex interplay between sociodemographic factors, knowledge, beliefs, and utilization of palliative and hospice care among patients with advanced lung cancer. Addressing these disparities will require multifaceted approaches that prioritize cultural humility, education, and proactive referral processes to ensure equitable access to high‐quality supportive care services for all individuals affected by lung cancer. Our results identify some of the underlying mechanisms driving these disparities and therefore can be used to develop effective interventions to address them in future research.

## Author Contributions

**Megan C. Edmonds:** conceptualization (equal), methodology (equal), writing – original draft (equal), writing – review and editing (equal). **Melissa Mazor:** methodology (equal), writing – original draft (equal), writing – review and editing (equal). **Mayuri Jain:** formal analysis (equal), writing – review and editing (equal). **Lihua Li:** formal analysis (equal), writing – review and editing (equal). **Marsha Augustin:** writing – original draft (equal), writing – review and editing (equal). **José Morillo:** writing – original draft (equal), writing – review and editing (equal). **Olivia S. Allen:** writing – review and editing (equal). **Amina Avril:** writing – review and editing (equal). **Juan P. Wisnivesky:** conceptualization (lead), funding acquisition (equal), methodology (equal), writing – original draft (equal), writing – review and editing (equal). **Cardinale B. Smith:** conceptualization (equal), data curation (lead), formal analysis (equal), funding acquisition (lead), investigation (equal), methodology (equal), writing – original draft (equal), writing – review and editing (equal).

## Conflicts of Interest

The authors declare no conflicts of interest.

## Data Availability

The authors have nothing to report.
